# Role of Genetics in Diagnosis and Management of Hypertrophic Cardiomyopathy: A Glimpse into the Future

**DOI:** 10.3390/biomedicines12030682

**Published:** 2024-03-19

**Authors:** Mohammed Tiseer Abbas, Nima Baba Ali, Juan M. Farina, Ahmed K. Mahmoud, Milagros Pereyra, Isabel G. Scalia, Moaz A. Kamel, Timothy Barry, Steven J. Lester, Charles R. Cannan, Rohit Mital, Susan Wilansky, William K. Freeman, Chieh-Ju Chao, Said Alsidawi, Chadi Ayoub, Reza Arsanjani

**Affiliations:** 1 Department of Cardiovascular Medicine, Mayo Clinic, Phoenix, AZ 85054, USA; abbas.mohammedtiseer@mayo.edu (M.T.A.); babaali.nima@mayo.edu (N.B.A.); farina.juanmaria@mayo.edu (J.M.F.); mahmoud.ahmed3@mayo.edu (A.K.M.); pereyra.milagros@mayo.edu (M.P.); scalia.isabel@mayo.edu (I.G.S.); kamel.moaz@mayo.edu (M.A.K.); barry.timothy@mayo.edu (T.B.); lester.steven@mayo.edu (S.J.L.); cannan.charles@mayo.edu (C.R.C.); mital.rohit@mayo.edu (R.M.); wilansky.susan@mayo.edu (S.W.); freeman.william@mayo.edu (W.K.F.); alsidawi.said@mayo.edu (S.A.); ayoub.chadi@mayo.edu (C.A.); 2 Department of Cardiovascular Medicine, Mayo Clinic, Rochester, MN 55905, USA; chao.chiehju@mayo.edu

**Keywords:** hypertrophic cardiomyopathy, sarcomeric genes, next-generation sequencing, whole exome sequencing, phenocopies, cascade testing, longitudinal surveillance, gene therapy

## Abstract

Hypertrophic cardiomyopathy (HCM) is the most common inherited cardiomyopathy. It follows an autosomal dominant inheritance pattern in most cases, with incomplete penetrance and heterogeneity. It is familial in 60% of cases and most of these are caused by pathogenic variants in the core sarcomeric genes (*MYH7*, *MYBPC3*, *TNNT2*, *TNNI3*, *MYL2*, *MYL3*, *TPM1*, *ACTC1*). Genetic testing using targeted disease-specific panels that utilize next-generation sequencing (NGS) and include sarcomeric genes with the strongest evidence of association and syndrome-associated genes is highly recommended for every HCM patient to confirm the diagnosis, identify the molecular etiology, and guide screening and management. The yield of genetic testing for a disease-causing variant is 30% in sporadic cases and up to 60% in familial cases and in younger patients with typical asymmetrical septal hypertrophy. Genetic testing remains challenging in the interpretation of results and classification of variants. Therefore, in 2015 the American College of Medical Genetics and Genomics (ACMG) established guidelines to classify and interpret the variants with an emphasis on the necessity of periodic reassessment of variant classification as genetic knowledge rapidly expands. The current guidelines recommend focused cascade genetic testing regardless of age in phenotype-negative first-degree relatives if a variant with decisive evidence of pathogenicity has been identified in the proband. Genetic test results in family members guide longitudinal clinical surveillance. At present, there is emerging evidence for genetic test application in risk stratification and management but its implementation into clinical practice needs further study. Promising fields such as gene therapy and implementation of artificial intelligence in the diagnosis of HCM are emerging and paving the way for more effective screening and management, but many challenges and obstacles need to be overcome before establishing the practical implications of these new methods.

## 1. Introduction

Hypertrophic cardiomyopathy (HCM) is a disease with a high burden of morbidity and mortality and is the most frequent cause of sudden cardiac death (SCD) in young athletes [1]. It is the most common inherited cardiomyopathy and one of the most prevalent genetic cardiovascular diseases [2,3]. Its prevalence ranges from 1:200 to 1:500 adults, currently affecting more than 750,000 individuals in the United States [2]. HCM may be characterized by dynamic left ventricular (LV) outflow obstruction, diastolic dysfunction, myocardial ischemia, and myocardial fibrosis and remodeling that increase susceptibility to severe arrhythmias [4].

The clinical diagnosis of HCM in adult patients is typically established using any cardiac imaging technique that shows an end-diastolic wall thickness of ≥15 mm anywhere in the left ventricle, which cannot be explained solely by pressure overload states, and after ruling out any other conditions that may cause LV hypertrophy. A cutoff of 13–14 mm can be diagnostic with a positive family history or a positive genetic test [5,6]. For diagnosis in children, the threshold is adjusted for body size and growth. Therefore, a Z-score ≥2 standard deviations above the mean for a child with a positive family history or a positive genetic test is sufficient for diagnosis, whereas a Z-score >2.5 in asymptomatic children with no family history is appropriate for diagnosis [5,6].

HCM is classified depending on the presence of positive family history or positive genetic test into familial (60%) and non-familial HCM (40%) [7,8,9]. Most of the familial cases follow autosomal dominant inheritance with incomplete penetrance, variable expressivity, and heterogeneity [2,6]. The remainder of the familial cases include different patterns of inheritance or HCM in the context of syndromes or other genetic disorders, particularly in children [10].

## 2. Genetic Basis of HCM

Most of the HCM positive-genetic test cases are caused by pathogenic variants (PV) in the sarcomeric genes, referred to as sarcomere-positive HCM (Table 1) [2]. Myosin heavy chain 7 (*MYH7*) and myosin-binding protein C3 (*MYBPC3*), encoding β-myosin heavy chain and myosin-binding protein C, respectively, are the two most common causal genes and are responsible for approximately 40% of all HCM cases and up to 70% of familial cases [11]. Other key sarcomeric genes that have robust evidence for being implicated in HCM are called core sarcomeric genes and along with the two aforementioned genes are: troponin T2 (*TNNT2*), troponin I3 (*TNNI3*), myosin light chain 2 (*MYL2*), myosin light chain 3 (*MYL3*), tropomyosin 1 (*TPM1*), and actin alpha cardiac muscle 1 (*ACTC1*) [6]. Other sarcomeric genes that have strong evidence of being implicated are filamin c (*FLNC*) and alpha kinase 3 (*ALPK3*) [12,13]. Troponin c1 (*TNNC1*), Cysteine and glycine-rich protein 3 (*CSRP3*), and actinin alpha2 (*ACTN2*) are also sarcomeric genes that have been reported to be involved in the pathogenesis of HCM, but with moderate evidence of causality [3].

Sarcomere-negative HCM includes conditions caused by mutations in non-sarcomeric genes and phenocopies. This includes genes that encode proteins that contribute to calcium handling, integrating the Z-disk and intercellular junctions such as phospholamban (*PLN*), which has strong evidence of causality, and junctophilin2 (*JPH2*) and formin homology2 domain-containing 3 (*FHOD3*), which have moderate evidence of causality [2,12,13]. Many other genes have been associated with HCM but with limited evidence of association; therefore, more studies and data are needed to establish the causality. Some of these genes are more associated with other cardiomyopathies such as *MYPN* and *NEXN*, which have shown an association with dilated cardiomyopathy [14,15]. Additionally, it is proposed that these genes may be the cause of HCM in cases where the standard genetic testing is negative. These genes with limited evidence include both sarcomeric genes such as (*TTN*, *MYH6*, *NEXN*, *TCAP*), and non-sarcomeric genes (*RYR2*, *VCL*, *MYOZ2*, *FHL2*) [2,5].

Interestingly, it has been revealed that genes associated with channelopathies such as *RYR2*, *KCNQ1*, and *DSP* may also cause HCM, but this association is not supported with enough evidence. Furthermore, it was proposed that epigenetic and environmental factors interact with the channelopathies-related genes to yield the HCM phenotype [16].

Nonetheless, according to the guidelines, only genes with strong evidence of association should be tested. The inclusion of the aforementioned genes in current panels could be considered, but more studies are needed to establish the validity of such practice [5,6].

A subset of HCM patients have mutations in other genetic loci that cause similar phenotypes to HCM but with the presence of syndromic, atypical features or other manifestations of genetic disorders that might be subtle and not apparent [13,17]. These conditions are known as phenocopies. The most relevant phenocopies are: Fabry disease (caused by PVs in *GLA*), familial amyloidosis (caused by PVs in *TTR*), Friedreich ataxia (caused by PVs in *FXN*), Noonan syndrome and other RASopathies (caused by PVs in genes in the Ras/mitogen-activated protein (MAP) kinase pathway), Danon disease (caused by PVs in *LAMP2*), Pompe disease (caused by PVs in *GAA*), desminopathy (caused by PVs in *DES*), and mitochondrial diseases (caused by PVs in mitochondrial genome) [7,8,13]. Sarcomere-negative HCM also includes conditions in which the genetic panel testing has not revealed any mutations, and with the absence of family history of the disease, this is termed as non-familial HCM [17].

Copy number variation (CNV) refers to a situation in which the number of copies of a particular DNA segment varies among individuals within a population. CNVs are a rare cause of inherited heart diseases associated with SCD, including HCM [18,19]. By using exome sequencing data, Singer et al. reported CNVs in genes strongly associated with HCM such as *MYH7* and *MYBPC3.* This highlights the feasibility of investigating these variations when standard genetic testing reveals no result [19].

In recent years, there has been significant advances in next-generation sequencing (NGS) as well as increased availability and decreased costs of whole genome sequencing (WGS) and whole exome sequencing (WES) techniques [8]. This has led to an expansion in the panel of genes known to be involved in HCM pathogenesis and to a better understanding of non-familial HCM. For the latter, there is growing evidence that it follows a complex pattern of inheritance, suggesting polygenetic inheritance in combination with environmental effects and epigenetic factors, which modify the expression of the phenotype [2,20,21]. In a multicenter retrospective cohort study including 400 familial and non-familial HCM patients and comparing risk factors prevalence between familial and non-familial HCM patients, it has been revealed that hypertension and obesity were significantly more prevalent in non-familial HCM patients, thus suggesting a role of environmental factors on the development of this phenotype [9]. Furthermore, two retrospective cohort studies have shown that obesity significantly potentiates HCM and is associated with worse clinical outcomes [22,23]. Moreover, environmental factors, such as intense training, and gene modifiers may increase the risk of clinical manifestations especially during exercise or sport [24].

## 3. Genetic Testing for HCM

In any patient with suspected HCM, a comprehensive medical history and physical examination and complete three-generation family history is recommended. If the clinical findings are suggestive of HCM, this first evaluation should be followed by electrocardiography (ECG) and cardiac imaging, usually with echocardiography initially [5,6]. In patients fulfilling the diagnostic criteria of HCM, genetic testing that at least includes the eight core sarcomeric genes which have the strongest evidence of disease causing (*MYBPC3*, *MYH7*, *TNNT2*, *TNNI3*, *TPM1*, *ACTC1*, *MYL2* and *MYL3*) is recommended in the patient (proband) according to the American Heart Association/American College of Cardiology (AHA/ACC) and European Society of Cardiology (ESC) guidelines. Also, phenocopies can be included in the first-tier genetic testing if there are clinical manifestations suggestive of syndromes or metabolic disorders, especially those conditions which have available treatments [5,6]. 

The importance of genetic testing stems from its role in identifying the molecular etiology of the disease, clarifying the diagnosis in some borderline cases, and distinguishing HCM from phenocopies, which has important clinical implications. Defining genetics may also help in guiding reproductive decisions, providing prognostic information, and guiding family screening and management [5,10,25]. A paramount step before and after genetic testing is genetic counselling, which involves trained health professionals such as genetic counsellors, genetic nurses, and/or medical geneticists with appropriate expertise [26]. Genetic counselling includes obtaining a detailed three-generation family history and depicting it on a pedigree, helping patients and their families to better understand and adapt to the medical and psychosocial consequences of their disease, providing clear explanations of genetic test findings, and discussing the anticipated results, goals, benefits, and limitations of the test. Genetic counselling prior to screening of relatives is critical as implications for insurance coverage if a test returns positive need to be discussed [27].

### 3.1. Genetic Testing Techniques

NGS techniques have revolutionized testing allowing high-throughput sequencing capability and decreased costs and timelines. This in turn has made the use of genetic testing for HCM widely available and easy to interpret and employ in clinical practice [28]. NGS includes target sequencing in customized disease-specific panels, WES and WGS, and it acts by the following basic steps: (1) fragmentation of DNA into regions of a few hundred base pairs (bp), (2) ligating adapter sequences on both ends of genomic fragments for library generation, (3) enrichment of targeted regions of interest by multiplexed PCR-based methods or by in-solution oligonucleotide hybridization-based methods that capture baits with streptavidin beads in solution, (4) generating strings of bases called “reads” by parallel sequencing, and (5) realigning and mapping the “reads” to the reference genome [29,30].

At present, targeted disease-specific panels that utilize NGS and include sarcomeric genes with the strongest evidence of association and syndrome-associated genes if there is clinical suspicion for phenocopies are the most used approach for genetic testing [10,31]. DNA for the test can be obtained from blood, saliva, or previously banked tissue. The yield of genetic testing for a disease-causing variant is 30% in sporadic cases and up to 60% in familial cases and in younger patients with typical asymmetrical septal hypertrophy [32].

Multiple studies have tried to improve the diagnostic yield of the test by implementing WGS and WES or by expanding the gene panels. A study including 58 unrelated patients with HCM (46 of them had a prior inconclusive genetic test) revealed that WGS detected a PV/LPV in 20% (9 out of 46) of cases in which a prior genetic test was inconclusive. Three subjects had a PV in genes (RASopathies genes and *CACNA1C*) that was not included in previous testing, five had a PV in non-coding regions including four with deep intronic variants in *MYBPC3* gene, and one had a PV in the mitochondrial genome [33].

Another study that included 41 HCM patients who had previous targeted HCM genetic testing showed no differences in diagnostic yield between WGS and targeted genetic testing. However, WGS identified one PV and variants of uncertain significance (VUS) in genes implicated in HCM but not included in targeted genetic testing and secondary genetic findings unrelated to the disease that may trigger further unnecessary testing [34]. Therefore, further studies are needed before establishing the validity of WGS and WES as first-tier tools for genetic testing in clinical practice as the benefits of increasing the diagnostic yield, inclusion of more genes, and discovering new variants that can be pathogenic should be balanced with the risks of false positive associations, over-reporting of uncertain findings, and the need for high expertise to interpret the large extracted dataset [35].

Artificial intelligence (AI) is revolutionizing the field of medical diagnostics, offering new and powerful tools for understanding complex genetic disorders such as HCM. Genetic testing can aid in developing family screening strategies and providing valuable diagnostic and prognostic information in HCM. However, it may also have a significant psychological impact. Therefore, patients may benefit from assessing their likelihood of having a sarcomeric mutation in HCM prior to genetic testing [36]. The Toronto HCM Genotype Score and the Mayo HCM Genotype Predictor were developed to determine patients likely to show positive genetic testing results for HCM. These scores consider clinical characteristics including the age of diagnosis, the greatest LV wall thickness, and a history of HCM in the family. These traditional scoring systems, however, have limited ability to predict genotype positivity and may be enhanced with the use of AI [36,37,38].

One study aimed to develop a new predictive model for genotype positivity using machine learning algorithms in HCM patients. This study showed that the machine learning model exhibited a greater capability of predicting the presence of genotype positivity in patients with HCM, outperforming the traditional Toronto and Mayo scoring systems [36]. Another study showed that a deep learning model based on non-enhanced cine cardiac magnetic resonance images and the Toronto score yielded significantly higher diagnostic performance in detecting HCM mutations [39].

A recent study used a convolutional neural network (CNN) to automatically identify features from ECGs that might not be noticeable to humans or through standard automated methods. The AI tool seemed to determine ECG features that were highly predictive of HCM [40].

AI algorithms and machine learning techniques are increasingly employed to analyze genetic data, identify patterns, and predict risks associated with HCM. This integration of AI in genetics and diagnosis is enhancing the ability to identify HCM early and accurately with non-invasive simple methods that can minimize numerous and costly tests for the patient, paving the way for personalized treatment strategies and improved patient outcomes [40].

### 3.2. Which Genes Should Be Looked for?

According to current guidelines, panels are generally limited to sarcomeric genes with the strongest evidence of association with the consideration of phenocopies only when there is clinical suspicion to minimize the risk of inconclusive findings [6]. The expansion to larger panels is emerging; however, it is not currently recommended because it does not provide clear benefits or additional diagnostic value over the currently used panels [41,42].

In a retrospective genetic database analysis for 1731 unrelated HCM patients who underwent genetic testing for at least one gene related to HCM phenocopies, 1.45% of subjects had a PV or LPV in one of these genes. Importantly, some of these patients had minimal or no extracardiac manifestations of the genetic disorder, and the identification of the disease changed the management of the patient. This highlights the utility of including phenocopies in genetic panels routinely [25].

A study using a systematic approach to assess the validity of reported gene–disease associations revealed that including genes with less supportive evidence of association should be considered carefully, because most of the variants identified were of uncertain significance and this has a negative impact on the patient and their family [13]. Another study including 1198 genetic testing results and comparing the diagnostic yield for three different genetic panels showed that in the clinical setting, only genes with definitive evidence of association yield actionable and interpretable results and expanding the genetic panel has no clinical benefit [41].

Another study, which included 2912 unrelated individuals with HCM, revealed that expanded gene panels encompassing more than 50 genes did not have additional clinical sensitivity compared to the original panels [42]. On the other hand, a study of 10 different families with non-syndromic pediatric HCM with negative test results who underwent genetic testing with an expanded gene panel identified PVs in five cases in genes that were not included in standard panels. This study proposed that expanded gene panels testing, or exome sequencing, might be beneficial in identifying the molecular diagnosis in pediatric patients when the initial genetic test fails [43].

### 3.3. Challenges of Genetic Testing and Classification of Variants

The most challenging and critical aspect of genetic testing is interpretation of results and classification of variants, depending on their association with the disease (benign (BV), likely benign (LBV), VUS, LPV, PV). Accuracy at this stage is critical, because misclassification of a variant as pathogenic may lead to overtesting and unnecessary concerns and, subsequently, psychosocial impacts on patients and their family. Alternatively, misclassification of a variant as benign can lead to false reassurance and missing the diagnosis of a serious disease. Therefore, this risk should be minimized by involving a multidisciplinary team in patient care including a genetic counsellor, clinical psychologist, cardiologist, allied health professionals, forensic pathologist, clinical geneticist, and nursing expert [8,44].

Moreover, the complexity of variant classification stems from the necessity of high expertise in human genetic variation and the limited and ambiguous data available [3,27]. To further minimize the risk of variant misclassification, The American College of Medical Genetics and Genomics (ACMG) in 2015 established the guidelines and standards that should be followed to classify and interpret the variants identified in a genetic test for a mendelian disease based on eight different types of evidence [45]. Moreover, the ACMG recommended that genetic testing should be conducted in a Clinical Laboratory Improvement Amendments-approved laboratory, and results should be interpreted by a board-certified clinical molecular geneticist or molecular genetic pathologist or equivalent [45].

The importance of expertise is highlighted in the following two studies. A retrospective variant re-analysis for 487 carriers of 530 genetic variants using the current ACMG criteria found that 22% of the variants were reclassified into different categories. Most importantly, 53 variants originally classified as PV/LPV were reclassified as VUS [46]. Another study of 136 unrelated HCM patients, in which 63 of them carried at least one mutation related to HCM, reassessed the pathogenicity of variants recognized in these patients based on the established criteria together with updated co-segregation data and population-based allele frequencies. The study reclassified 105 of the variants [47].

Considering these findings, and the rapid expansion of genetic knowledge, it is necessary to reassess the classification of variants every few years and follow-up with patients regarding their results. If the variant has been upgraded to PV/LPV, family screening can be approached, or if the variant has been downgraded to BV/VUS, family members should undergo longitudinal clinical screening. Consequently, the ACMG has recommended specific protocols and criteria that should be followed by clinical laboratories regarding variant reclassification and these criteria should keep pace with any new developments in population databases, genomic data, bioinformatics, and approaches and resources in genetic results interpretation [48,49].

As knowledge increases and genetic techniques improve, more resources and approaches have been developed to improve results interpretation and classification of variants. The most significant database resources in this domain include: CardioClassifier, Clinical Genome Resource (ClinGen), ClinVar, and the Genome Aggregation Database (gnomAD) [8,44]. A unique quantitative approach has been shown to increase the classification of variants into clinically actionable results by 14–20%. This approach has used the etiological fraction and a dedicated unsupervised clustering algorithm to improve the diagnostic yield of the genetic test and interpretation of results [50]. Additionally, ClinGen’s inherited cardiomyopathy expert panel (CMP-EP) has applied modified ACMG criteria in combination with expert review and clinical judgement to improve the classification of *MYH7* variants. This has increased the specificity of *MYH7* variant classification in *MYH7*-associated disorders [51].

## 4. Role of Genetic Testing in Guiding Family Screening and Management

**Figure 1:**
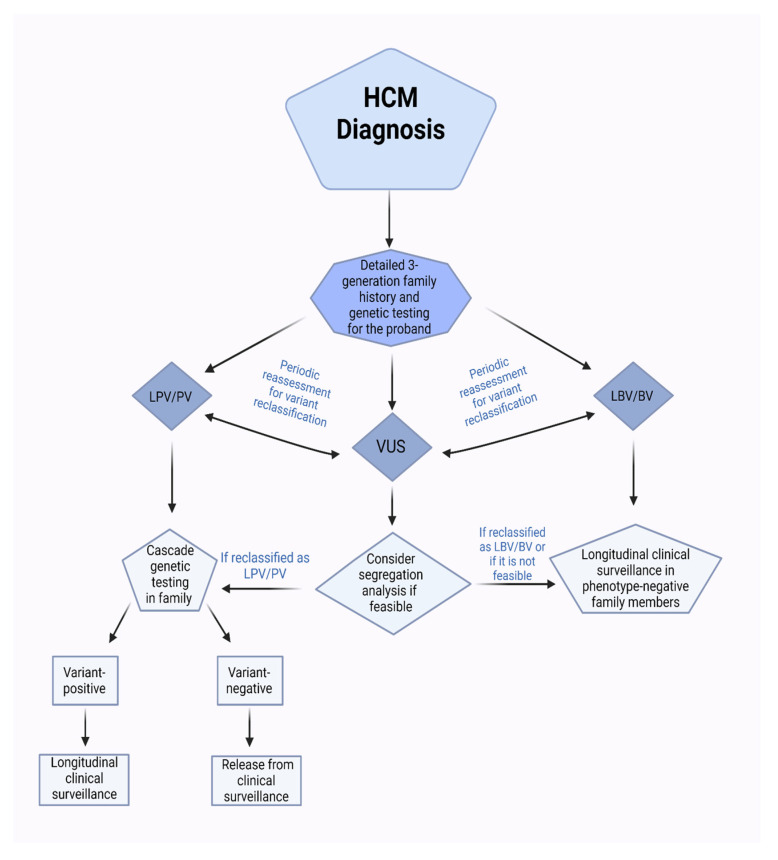
Flowchart illustrates genetic testing and screening in HCM. HCM: Hypertrophic cardiomyopathy; LPV/PV: likely pathogenic variant/pathogenic variant; VUS: variant of unknown significance; LBV/BV: likely benign variant/benign variant.

Genetic testing for HCM includes two components: diagnostic testing for the individual patient to confirm the HCM diagnosis and identify a PV, and then predictive (cascade) testing in first-degree relatives (Figure 1) [52]. Current guidelines recommend focused cascade genetic testing regardless of the age in phenotype-negative first-degree relatives if a variant with decisive evidence of pathogenicity has been identified in the proband, with emphasis on the importance of periodic reassessment of variant classification regarding pathogenicity [5,6]. The result of the cascade test guides further management in at-risk family members. A negative test result for the disease-causing mutation identified in the proband ‘rules out’ HCM, and further clinical (longitudinal) surveillance is not required unless new signs or symptoms of the disease develop. On the other hand, family members who have the disease-causing mutation identified in the cascade testing need lifelong clinical surveillance. In case of a negative genetic test in the proband or if the genetic test was not performed for any reason, cascade genetic testing is not recommended; however, first-degree relatives should be screened clinically using ECG and echocardiography for HCM. If there is no evidence of the disease, they should follow longitudinal clinical surveillance with serial clinical assessment, ECG, cardiac imaging, and additional appropriate tests depending on clinical judgement. Longitudinal clinical surveillance is typically performed at regular intervals depending on age: every one to two years in children and adolescents, and every three to five years in adults (AHA/ACC), or every one to three years before the age of 60, and then every three to five years thereafter (ESC) [5,6].

Previously, there was controversy regarding cascade genetic testing and clinical screening in first-degree relatives of the proband aged less than 10 years, in the absence of family history of premature death from HCM, participation in competitive sports, or any clinical aspect that suggests early HCM [53]. It is now recommended to test them regardless of their age or risk factors according to recent data revealing that a significant percentage of HCM patients can manifest before 10 years of age with high morbidity and mortality regardless of their risk factors [5,6]. Additionally, the data showed the clinical significance and the minimum negative impacts of testing on the child in the context of multidisciplinary care that includes psychosocial support [53].

A large single-center study of 1198 consecutive children aged 18 years or less with a first-degree relative with HCM revealed that serial evaluation identified 32 individuals (at baseline) and 25 individuals (during follow-up) who met the diagnostic criteria for HCM. Forty-four patients (72%) were younger than 12 years of age, and testing changed the clinical course of these patients [54]. Another retrospective cohort study of 524 subjects aged less than 18 years who had first-degree relatives with HCM and were screened clinically for HCM independent of age showed that one-third of the cases who were not eligible for early screening based on older guidelines met the diagnostic criteria for HCM and 76.5% of children who had major adverse cardiovascular events (MACE) had HCM that had been diagnosed before 10 years of age [55].

Any doubts regarding the pathogenicity of the variant identified in the proband, indicate that cascade genetic testing should not be pursued, and at-risk relatives should have regular surveillance evaluations with echocardiography. In selected cases, when a VUS has been found in the proband, testing family members for clinical or research purposes can be pursued to further clarify the pathogenicity of the variant. This can be achieved using co-segregation analysis in family members and functional studies with cardiovascular genetics expert supervision [8,56].

It is also recommended in cases of SCD with HCM diagnosis confirmed by autopsy to perform genetic testing (molecular autopsy) on the proband to determine if PV can be identified and subsequently cascade genetic testing may be pursued in family members for risk stratification. This is particularly important in instances where the family variant is unknown or no other affected family members are still alive [57]. A cross-sectional survey that identified family members affected by the SCD of one of their relatives experienced significant prolonged grief and post-traumatic stress [58]. This highlights the importance of genetic testing in stratifying the risk of SCD for at-risk relatives to relieve some of their concerns and decrease the risk of psychological morbidity [59].

Given the hereditary nature of HCM and its high morbidity and mortality, pre-conception and prenatal genetic counselling and testing should be offered when couples have a previously affected child, or one or both carry a known PV [5,6]. Prenatal genetic counseling is paramount for prospective parents to explain the risk of transmission of the disease to offspring and the available reproductive options. This should be performed as early as possible in pregnancy or before conception to give the parents a chance to make a fully informed decision. Prenatal genetic diagnosis includes chorionic villus sampling, amniocentesis, non-invasive prenatal testing, and fetal echocardiography (for selected families) [17]. Preimplantation genetic diagnosis is an established clinical procedure in which in vitro fertilization (IVF) is used to obtain 3-day stage embryos and then genetic testing is performed on each embryo to determine the ones that do not carry the mutation, so they can be transferred into the uterus to initiate pregnancy [60].

## 5. Role of Genetic Testing in Predicting Prognosis and Guiding Management of HCM

**Figure 2:**
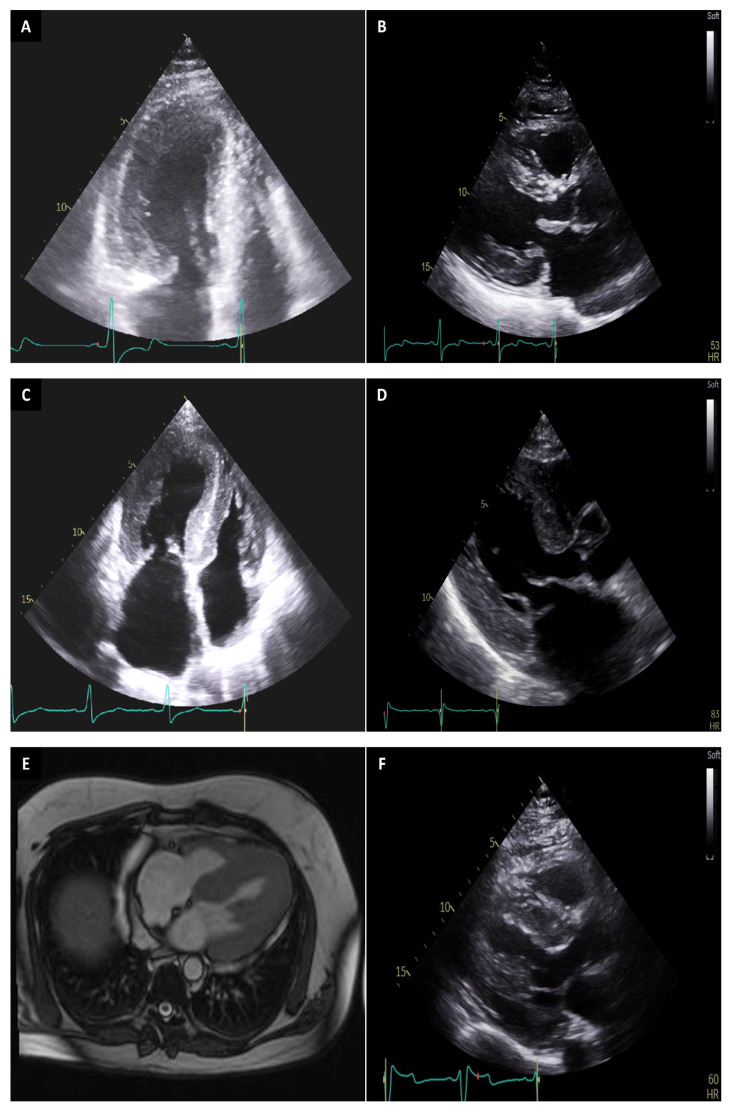
Cardiovascular imaging revealing hypertrophic cardiomyopathy (HCM) and phenocopies. Panel A and B show apical 4-chamber (**A**) and parasternal long axis (**B**) transthoracic echocardiography (TTE) views demonstrating asymmetric septal hypertrophy (reverse curve morphology, maximal wall thickness 19 mm) in a patient with confirmed typical HCM. Panel C and D depict apical 4-chamber (**C**) and parasternal long axis (**D**) TTE views showing increased wall thickness of the left and right ventricle and enlarged left atrium in a patient with light chain (AL) amyloidosis. Panel E and F show images from a patient initially diagnosed with HCM; however, genetic testing confirmed the diagnosis of Fabry’s disease in the absence of any other clue for Fabry; magnetic resonance imaging coronal section (**E**) reveals diffuse left ventricular hypertrophy and parasternal long axis TTE view (**F**) shows increased wall thickness.

While there is established evidence and guidelines for the use of genetic testing in diagnosis and screening of HCM, its evidence for prognostic stratification and treatment guidance is still limited, and further studies are needed to establish its use in this regard. Currently, there is evolving evidence for the integration of genetic testing results in risk-stratification algorithms and informing management [7,61]. One established role for genetic testing in management is differentiating primary HCM from HCM mimics in cases that lack extracardiac manifestations (Figure 2). This may completely change the management of the patient, especially for some syndromes with well-established therapies. For example, enzyme replacement therapy in Fabry disease can reverse LV hypertrophy and Danon syndrome usually needs aggressive clinical management and possibly transplant [62]. MEK-inhibition via trametinib significantly reduced LV hypertrophy and NT-proBNP levels in Noonan Syndrome [63]. Multiple studies have shown that patients with negative genetic testing and no family history tend to manifest in older age, have less lifetime risk of death, have a benign clinical course, and have less major cardiac events compared to genotype-positive patients. This in turn highlights the importance of more aggressive management for patients with positive genetic testing [9,64,65,66].

One study examining the significance of multiple rare variants in 758 HCM patients revealed that those who carried multiple variants had worse all-cause mortality rates [67]. A single-center retrospective cohort study including 1243 patients with HCM (125 of them with familial HCM) revealed that a positive genetic test was an independent prognostic factor for cardiovascular mortality and cardiac transplantation in HCM patients [68]. Moreover, some studies have suggested that genetic heterogeneity underlies the variability in phenotype and severity of the disease, as disease course and outcomes differ depending on the implicated genes and even on the variant in the same gene [32]. For example, patients with PV in *MYH7* have a higher incidence of atrial fibrillation, earlier age of onset, and more severe disease compared to those who carry PV in the *MYBPC3* gene [69,70]. Also, *TNNI3* mutation carriers have lower survival than *MYBPC3* and *MYH7* mutation carriers, and *TNNC1* mutation carriers have a higher risk of developing fatal ventricular arrhythmias [71]. A study describing a new truncating mutation in the *MYBPC3* gene (p.Val931Glyfs*120) revealed that this variant is associated with high penetrance and higher risk of SCD in men, requiring implantable cardioverter defibrillator (ICD) placement [72]. Moreover, those with splice donor mutations in the *MYBPC3* gene were more susceptible to ventricular arrhythmias (even before the phenotypical HCM manifests) compared to those with frameshift and nonsense mutations [73,74].

Another study of 283 patients compared the current risk-stratification models for SCD and the need of ICD implantation used by guidelines with a modified risk prediction tool integrating genetic findings. This study revealed a sensitivity of 0.86 and specificity of 0.69 for the modified tool compared to 0.93 and 0.28 for the AHA/ACC model, and 0.29 and 0.83 for the ESC model. This in turn highlights the feasibility of this tool to be utilized in decision making regarding management of HCM patients [75].

Thus, a precision medicine-based approach that involves genetics in the management of HCM can be further assessed in future studies to establish the integration of genetics in management and the adoption of personalized treatment plans depending on the patient genotype and clinical factors [76].

There is conflicting data regarding the risk of SCD and arrhythmias and genetic status. Thus, the placement of ICD for primary prevention in phenotype-negative genotype-positive patients at this time is generally not guided by the type of gene identified. Molecular changes in cardiac cells that predate hypertrophy and gross detection of the disease can make the cardiac tissue inherently abnormal and arrhythmogenic, and recent case studies have reported non-fatal arrythmias in phenotype-negative genotype-positive patients [77,78]. However, one study of 1660 patients including patients with no LV hypertrophy and positive genotype has shown that intensive exercise was not associated with higher rates of death or serious arrhythmias compared to moderate exercise or sedentary lifestyle [79]. This has led to uncertainty about participation in competitive sports and placement of ICD as primary prevention in these patients. At present, given the low risk of SCD in these patients, they are not restricted from competitive sports unless there is a family history indicating a high risk of SCD. Similarly, ICD placement for primary prevention is not recommended. Further work is needed to better understand the course of the disease in these patients and their eligibility for ICD placement [6,78].

## 6. Gene Therapy and Future Directions

**Figure 3:**
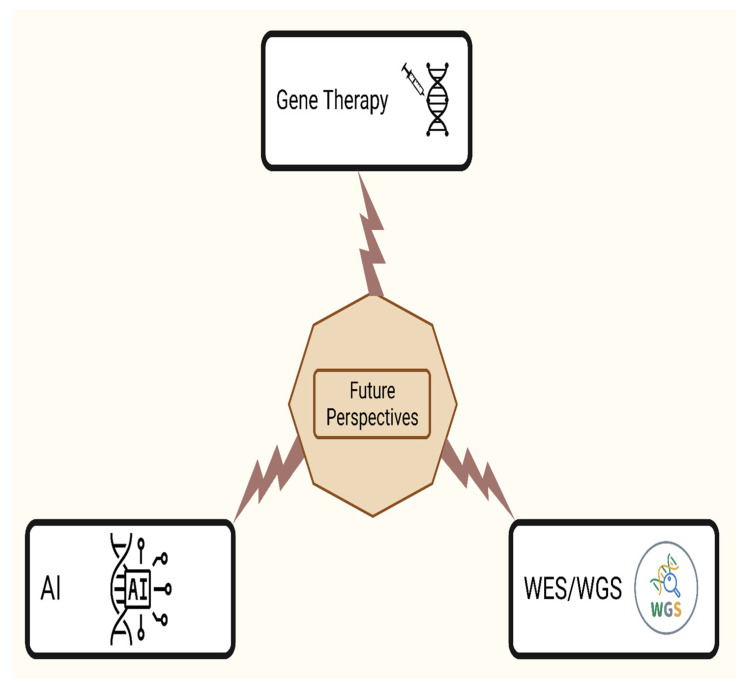
Illustration of future directives and evolving fields in employing genetics in HCM management. HCM: hypertrophic cardiomyopathy; AI: artificial intelligence; WES/WGS: whole exome sequencing/whole genome sequencing.

AI and WES/WGS (already discussed) and gene therapy are active research areas that constitute future directions (Figure 3). However, further studies are needed to establish their value in clinical practice. Furthermore, the integration of genetic testing into risk-stratification algorithms and management of phenotype-negative genotype-positive patients still lack sufficient evidence to be practically implemented [80]. The evolving understanding of the genes involved in HCM has improved the screening for this condition and the development of new treatments that directly target the molecular pathogenesis of the disease. Since HCM can be caused by different dominant missense or loss-of-function (LoF) mutations in sarcomere genes, a straightforward way to prevent the disease could be to fix each harmful variant before symptoms manifest [81].

A novel study tested the strategy of fixing the DNA in gametes before fertilization, also known as gene editing. The sperm had a small error in the *MYBPC3* gene, while the egg had the normal variant. They used CRISPR/Cas9 gene editing technology to fix the error in the *MYBPC3* gene in the sperm. In the experiment, when the CRISPR reagents were added 18 h after fertilization, 33% of the embryos still had both the normal and the faulty *MYBPC3* gene, or were mosaics, meaning they had a mix of corrected and mutant cells. If the CRISPR reagents were injected at the same time as the sperm, 72% of the embryos ended up with two normal copies of the *MYBPC3* gene and showed no signs of mosaicism. While gene-based embryo screening can prevent HCM, the practical use of these gene correction methods poses significant ethical, legal, and social concerns [82].

Some preclinical treatments for HCM in mice focus on inducing specific gene changes directly in vivo and modifying the abnormal gene, also known as gene replacement. One study evaluated two different genetic therapies—an adenine base editor (ABE8e) and a potent Cas9 nuclease—both delivered via AAV9 (Adeno Associated Virus serotype 9) to prevent disease in mice with heterozygous HCM PV, myosin R403Q. Administering a single dose of dual-AAV9 vectors, each containing one part of the RNA-guided ABE8e, successfully corrected the PV in over 70% of ventricular cardiomyocytes and sustained normal cardiac structure and function [83]. Gene therapy with AAV9 has demonstrated successful long-term treatment in homozygous *MYBPC3*-targeted knock-in (KI) mice [81]. A single systemic administration of AAV9-*MYBPC3* in 1-day-old KI mice prevented the development of cardiac hypertrophy and dysfunction during the observation period of 34 weeks and increased *MYBPC3* messenger RNA (mRNA) and *MYBPC3* protein levels in a dose-dependent manner [84].

In many instances of HCM, there is an issue with calcium regulation within the heart cells. Calcium is vital not only for excitation–contraction coupling but also acts as a signal that can lead to hypertrophic remodeling [85].

A key player in controlling calcium in heart cells is a protein called SERCA2a (sarcoplasmic reticulum calcium-ATPase 2a). It helps quickly move calcium back into the sarcoplasmic reticulum. This process is slowed down by another protein, PLN. To improve diastolic dysfunction, a common problem in HCM, one strategy is to change the balance of SERCA2a and PLN. This can be conducted by either reducing the amount of PLN or increasing the amount of SERCA2a. AAV-mediated delivery of the *SERCA2a* gene into newborn transgenic mice that overexpressed a human HCM missense variant in α-tropomyosin showed promising results. This treatment reduced hypertrophy and fibrosis, and it also normalized heart function compared to untreated mice [85]. Another method involved removing *PLN* by breeding mice that naturally lack *PLN*. This increased the activity of SERCA2a, which helps the heart muscle manage calcium better. Mice with both the α-tropomyosin mutation and no *PLN* had hearts of normal size, less collagen buildup, and improved heart function. However, applying similar strategies in humans might be challenging. While removing *PLN* can help with HCM, having one less copy of the *PLN* gene (heterozygous loss-of-function variants) in humans can lead to a different heart condition called dilated cardiomyopathy (DCM), making this approach less feasible for human treatment [85].

TN-201 is a pioneering gene therapy under investigation, using an AAV vector to target HCM caused by mutations in the *MYBPC3* gene. This first-of-its-kind treatment aims to introduce a functional *MYBPC3* gene directly into the heart’s cells through a one-time infusion, tackling the root cause of HCM associated with *MYBPC3* gene defects. TN-201 is currently being evaluated in the MyPeak-1, which is a Phase 1b multicenter clinical trial, to assess the tolerability and safety of this therapy in six patients diagnosed with *MYBPC3*-associated nonobstructive HCM. This is the first trial that tests gene therapy in humans in the hopes of developing an established targeted therapy that can cure the disease in the future [86].

Although promising, the application of gene therapy in HCM still requires extensive clinical trials. There is a considerable journey ahead before these therapies can be reliably and safely used in clinical settings.

As mentioned, the management of phenotype-negative genotype-positive patients is under discussion. Early intervention targeting calcium (Ca^2+^) regulation in this subgroup of HCM patients might prevent pathological hypertrophy and enhance cardiac function, especially in cases that exhibit increased myofilament sensitivity to Ca^2+^ and diastolic dysfunction [87]. A pilot randomized trial suggested that diltiazem administration in genotype-positive carriers without LVH may improve early LV remodeling in HCM. Interestingly, individuals with the *MYBPC3* mutation might respond better to treatment with diltiazem compared to those with the *MYH7* mutation. *MYBPC3* mutation carriers who received diltiazem showed significantly less increase in LV wall thickness and mass over time [88].

Mavacamten is a novel first in-class cardiac myosin inhibitor. Specifically, it reduces excessive cross-bridging with actin, which is believed to be an important contributor to the pathological hypercontractility associated with HCM caused by PVs in *MYH7* and *MYBPC3* that affect the normal relaxation of myosin [89,90]. It is specifically prescribed for adults with symptomatic obstructive HCM to enhance functional capacity and alleviate symptoms. Mavacamten received approval from the US Food and Drug Administration (FDA) in April 2022 following the successful completion of the EXPLORER-HCM trial [91]. Genetic status may also predict the response to mavacamten as genotype-positive patients have a dramatic response compared to genotype-negative patients.

Another recent myosin inhibitor, designed for obstructive HCM patients, represents a new advancement in this field. This drug, Aficamten, stands out for its shorter half-life and reduced interactions with other medications compared to Mavacamten [92]. Findings from a phase II study revealed that Aficamten effectively decreased the left ventricular outflow tract (LVOT) gradient and improved heart failure symptoms in patients with obstructive HCM compared to Mavacamten [93].

## 7. Conclusions

The integration of genetics into HCM diagnosis and management has been evolving rapidly in recent years. At present, diagnostic genetic testing and cascade genetic testing are critical in clinical practice, with limitations regarding diagnostic yield and interpretation of results. These may be improved with the expansion of genetic panels, implementing WES and WGS in testing, and developing effective tools for classification of the variants that keep up with rapid genetics development. There is a promising role for implementation of genetic testing results in risk stratification and guiding management for HCM; however, more studies are needed to better integrate it into clinical practice. The role of AI in prediction of genetic test results before testing and gene therapy approaches are promising and evolving fields related to HCM genetics.

## Figures and Tables

**Table 1:** Genes involved in HCM pathogenesis.

	Gene Abbreviation	Gene Name	Encoded Protein	Mode of Inheritance	Clinical Validity	% of Familial HCM Cases Caused by PVs in the Gene
Core Sarcomeric Genes	*MYBPC3*	Cardiac myosin-binding protein C	Cardiac myosin-binding protein C	AD, AR	Definitive	40–45
	*MYH7*	Myosin heavy chain 7	Cardiac β-myosin heavy chain	AD	Definitive	15–25
	*TNNI3*	Troponin I3	Cardiac troponin I	AD	Definitive	1–7
	*TNNT2*	Troponin T2	Cardiac troponin T	AD	Definitive	1–7
	*TPM1*	Tropomyosin 1	α-tropomyosin	AD	Definitive	1–2
	*MYL2*	Myosin light chain 2	Myosin regulatory light chain	AD	Definitive	1–2
	*MYL3*	Myosin light chain 3	Myosin essential light chain	AD, AR	Definitive	1–2
	*ACTC1*	Myosin light chain 2	α-cardiac actin	AD	Definitive	1–2
Other Sarcomeric Genes	*FLNC*	Filamin c	Filamin c	AD	Definitive	<1
	*ALPK3*	Alpha kinase 3	Alpha kinase 3	AR	Definitive	<1
	*TNNC1*	Troponin c1	Cardiac troponin c	AD	Moderate	<1
	*CSRP3*	Cysteine and glycine-rich protein 3	Muscle LIM	AD	Moderate	<1
	*ACTN2*	Actinin alpha 2	α-actinin	AD	Moderate	<1
	*TTN*	Titin	Titin	AD	Limited	<1
	*MYH6*	Myosin heavy chain 6	α-myosin heavy chain	AD	Limited	<1
	*MYPN*	Myopalladin	Myopalladin	AD	Limited	Rare
	*NEXN*	Nexilin	Nexilin	AD	Limited	Rare
	*TCAP*	Titin cap gene	Telethonin	AD	Limited	Rare
Non-Sarcomeric Genes	*PLN*	Phospholamban	Phospholamban (regulates calcium pump in cardiac myocytes)	AD	Definitive	<3
	*JPH2*	Junctophilin2	Junctophilin 2 (sarcoplasmic reticulum-surface membrane binding protein)	AD	Moderate	<1
	*FHOD3*	Formin Homology 2 Domain Containing 3	FHOD3 (a myocardial formin)	AD	Moderate	<1
	*RYR2*	Ryanodine receptor2	Ryanodine receptor2 (calcium-induced calcium release)	AD	Limited	Rare
	*VCL*	Vinculin	Vinculin/metavinculin (intercalated disk protein)	AD	Limited	Rare
	*MYOZ2*	Myozenin 2	Myozenin2 (Z-disk protein)	AD	Limited	<1
	*FHL2*	Four and a half LIM domains 2	Four and a half LIM domains 2	Non-Mendelian	Limited	Rare
	*ANKRD1*	Ankyrin repeat domain 1	Cardiac ankyrin repeat domain	Non-Mendelian	Limited	Rare
	*OBSCN*	Obscurin	Obscurin	AD	Limited	Rare
phenocopies	*LAMP2* (Danon disease)	Lysosome-associated membrane protein 2	Lysosome-associated membrane protein 2	XL	Definitive	Rare
	*GLA* (Fabry disease)	α-galactosidase A	α-galactosidase	XL	Definitive	Rare
	*TTR* (familial amyloidosis)	Transthyretin	Transthyretin	AD	Definitive	Rare
	*PTPN11* (Noonan syndrome)	Protein tyrosine phosphatase non-receptor type 11	Protein tyrosine phosphatase non-receptor type 11	AD	Definitive	Rare
	*RAF1*(Noonan syndrome)	*RAF-1* proto-oncogene	RAF serine/threonine kinase	AD	Definitive	Rare
	*RIT1* (Noonan syndrome)	*RIT1* gene	GTP-binding protein RIT1	AD	Definitive	Rare
	*PRKAG2* (PRKAG2 cardiomyopathy)	Protein Kinase AMP-Activated Non-Catalytic Subunit Gamma 2	AMP-activated protein kinase	AD	Definitive	Rare
	*DES* (Desminopathy)	Desmin	Desmin	AD/AR	Definitive	Rare
	*FHL1* (Emery-Dreifuss muscular dystrophy)	Four and a half LIM domains 1	Four and a half-Lim-only	XL	Definitive	Rare
	*LDB3* (Myofibrillar myopathy)	LIM domain binding 3	LIM domain binding 3	AD	Definitive	Rare
	*BAG3* (Myofibrillar myopathy)	Bcl2-associated athanogene 3	BAG Cochaperone 3	AD	Definitive	Rare
	*FXN* (Friedreich ataxia)	Frataxin	Frataxin	AR	Definitive	Rare
	*GAA* (Pompe disease)	Acid α-glucosidase	Lysosomal α-glucosidase	AR	Definitive	Rare
	*CACNAC1C* (Timothy syndrome)	Calcium voltage-gated channel subunit alpha1 C	Voltage-dependent calcium channel	AD	Definitive	Rare

AD: autosomal dominant; AR: autosomal recessive; XL: X-linked; PVs: pathogenic variants.

## Data Availability

No new data were created or analyzed in this study. Data sharing is not applicable to this article.
